# Enhanced Visual Search in Infancy Predicts Emerging Autism Symptoms

**DOI:** 10.1016/j.cub.2015.05.011

**Published:** 2015-06-29

**Authors:** Teodora Gliga, Rachael Bedford, Tony Charman, Mark H. Johnson, The BASIS Team, Simon Baron-Cohen, Patrick Bolton, Celeste Cheung, Kim Davies, Michelle Liew, Janice Fernandes, Issy Gammer, Helen Maris, Erica Salomone, Greg Pasco, Andrew Pickles, Helena Ribeiro, Leslie Tucker

**Affiliations:** 1 Centre for Brain and Cognitive Development, Birkbeck College, University of London, Malet Street, London WC1E 7HX, UK; 2 Department of Biostatistics, Institute of Psychiatry, Psychology and Neuroscience, King’s College London, London SE5 8AF, UK; 3 Department of Psychology, Institute of Psychiatry, Psychology and Neuroscience, King's College London, London SE5 8AF, UK

## Abstract

In addition to core symptoms, i.e., social interaction and communication difficulties and restricted and repetitive behaviors, autism is also characterized by aspects of superior perception [1]. One well-replicated finding is that of superior performance in visual search tasks, in which participants have to indicate the presence of an odd-one-out element among a number of foils [2–5]. Whether these aspects of superior perception contribute to the emergence of core autism symptoms remains debated [4, 6]. Perceptual and social interaction atypicalities could reflect co-expressed but biologically independent pathologies, as suggested by a “fractionable” phenotype model of autism [7]. A developmental test of this hypothesis is now made possible by longitudinal cohorts of infants at high risk, such as of younger siblings of children with autism spectrum disorder (ASD). Around 20% of younger siblings are diagnosed with autism themselves [8], and up to another 30% manifest elevated levels of autism symptoms [9]. We used eye tracking to measure spontaneous orienting to letter targets (O, S, V, and +) presented among distractors (the letter X; Figure 1). At 9 and 15 months, emerging autism symptoms were assessed using the Autism Observation Scale for Infants (AOSI; [10]), and at 2 years of age, they were assessed using the Autism Diagnostic Observation Schedule (ADOS; [11]). Enhanced visual search performance at 9 months predicted a higher level of autism symptoms at 15 months and at 2 years. Infant perceptual atypicalities are thus intrinsically linked to the emerging autism phenotype.

## Highlights

• We measured visual search abilities in infants at familial risk for autism
• Enhanced visual search at 9 months predicted a higher level of autism symptoms at 2 years
• Atypical perception is intrinsically linked to the emerging autism phenotype

## Teaser

It remained unknown whether superior perception, a common feature of the autism phenotype, contributes to the emergence of core social interaction and communication symptoms. Gliga et al. show that superior performance in a visual search task in 9-month-old infants predicts a higher level of autism symptoms at 15 months and 2 years.

## Results and Discussion

Eighty-two high-risk infants (37 girls) and 27 low-risk controls (13 girls) took part in this study (Table S1). We analyzed the proportion of trials in which infants made a first look toward one of the targets, after fixating at the center of the screen. Infants with at least four valid trials were included in the analysis. Above-chance performance was measured in the group as a whole, at all ages (9 months: t(103) = 5.62, p < 0.001; 15 months: t(95) = 4.31, p < 0.001; 2 years: t(94) = 7.9, p < 0.001; Table S2). Performance was not related to either the age or IQ of the participant (all p > 0.09) during any of the visits.

**Figure 1:**
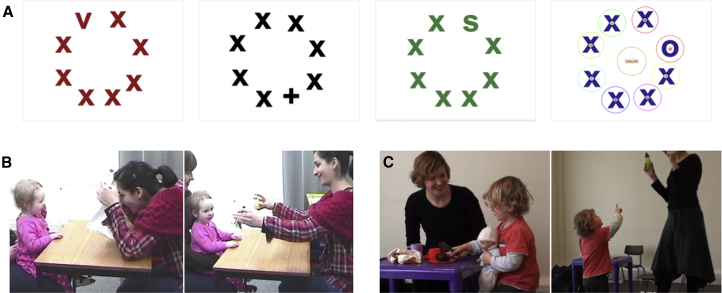
Study Design, Stimuli, and Behavioral Assessments (A) Example stimuli and the areas of interest (AOIs) used in analysis. (B) Example of behaviors assessed with the AOSI (e.g., anticipation of social contact, attention shifting). (C) Example of behaviors assessed with the ADOS (e.g., pretend play, pointing).

**Figure 2:**
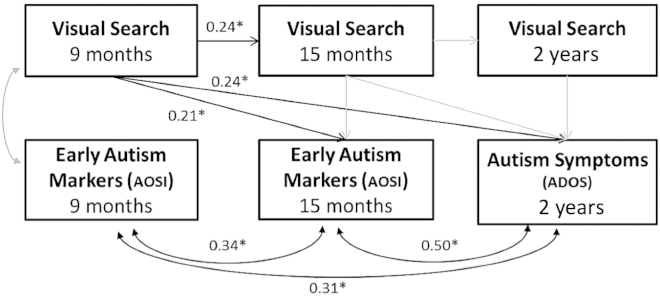
Relationship between Visual Search and Emerging Autism Symptoms Visual search performance at 9 months predicts later autism symptom severity in an autoregressive model. Standardized coefficient values are presented for significant results (represented as black arrows).

A shift from categorical to continuous characterization of psychopathology is encouraged by clinical and genetics research [12, 13]. Thus, to take into account longitudinal relationships between visual search performance and emerging autism symptoms (Figure 1), we entered search performance at 9 months, 15 months, and 2 years in an autoregressive model (Figure 2; model fit: χ^2^(4) = 6.87, p = 0.14, comparative fit index [CFI] = 0.95) with continuous measures of symptom severity at 9 and 15 months (Autism Observation Scale for Infants [AOSI] score) and 2 years of age (Autism Diagnostic Observation Schedule [ADOS] score). Nine-month visual search significantly predicted the 15-month AOSI (β = 0.22, SE = 0.10, p = 0.03) and the 2-year ADOS (β = 0.24, SE = 0.10, p = 0.02; see also Figure S2) score, with increased visual search accuracy predicting higher symptom severity. Findings were very similar when only the high-risk group was included in the analysis (model fit: χ^2^(4) = 10.001, p = 0.04, CFI = 0.88). The relationship with ADOS remained substantively similar: 9-month visual search was significantly related to both 15-month AOSI (β = 0.223, SE = 0.11, p = 0.049) and 2-year ADOS (β = 0.27, SE = 0.11, p = 0.02) scores. To test whether visual search at 9 months continued to predict ADOS score after accounting for earlier autism markers, we ran an autoregressive model with regressions, rather than correlations, between AOSI and ADOS (model fit: χ^2^(4) = 6.87, p = 0.14, CFI = 0.95; Figure S1). The relationship between 9-month visual search and 15-month AOSI remained significant (β = 0.182, SE = 0.09, p = 0.046), but the direct relationship with later ADOS (i.e., accounting for 9- and 15-month AOSI) became non-significant (β = 0.13, SE = 0.09, p = 0.13), suggesting a developmental pathway in which infant visual search contributes to autism symptoms at 15 months of age and that in turn contributes to autism severity at 2 years of age. The lack of a concurrent relationship between visual search and symptom severity at 9 months (β = 0.08, SE = 0.10, p = 0.44) is suggestive of a causal pathway from early perception to later emerging autism symptoms. Moreover, although 9- and 15-month visual search performances are correlated (β = 0.24, SE = 0.10, p = 0.02), performance at later time points, i.e., at 15 months and 2 years of age, does not relate to symptomatology. This differential relationship points to particular periods in early postnatal development within which atypical perception, in addition to other risk factors, may set development on a pathway to pathology and further highlights the importance of prospective studies of early development.

We demonstrate, for the first time, a relationship between superior visual search abilities during infancy and the severity of later autism symptoms. Because we analyzed the first saccade made in each trial, and not whether infants ever visited the target during the trial (as in some previous visual search studies [5]), we are confident our findings are not confounded by differences in oculomotor behavior described in this population [14]. Also, given a higher incidence of hyperlexia in autism [15], future studies should address the question of whether the demonstrated superior visual search also predicts better recognition of letters later in childhood.

Views on atypical perception have alternated between assigning it a core, causal role in autism [16] and portraying it as “one aspect of cognition in autism spectrum disorder (ASD) alongside, rather than causing/explaining, deficits in social cognition” [6]. Importantly, our findings corroborate evidence for atypical oculomotor behavior [14] and increased frontal-occipital functional connectivity [17] during the first year of life of those infants that later develop autism symptoms, by suggesting that perturbations in general processes, such as perception or attention, are more important than previously believed in the developmental pathway to this disorder [18]. With this shift away from “social brain” theories of autism (e.g., [19]) comes also the challenge of explaining the mechanisms through which domain-general atypicalities could contribute to the emergence of specific autism symptoms. Moreover, the striking predictive association between superior visual search and autism may also prove useful as one additional component of early autism identification, given a context in which most current infant markers are based on impairments common to multiple neurodevelopmental outcomes (e.g., [20–22]).

## Experimental Procedures

### Participants

Participants took part in a longitudinal study of children at risk for autism. At the time of enrollment, none of the infants had been diagnosed with any medical or developmental condition. Twenty-seven low-risk participants and 82 high-risk participants took part in this study. High-risk infants had at least one older sibling (hereafter, proband) with a community clinical diagnosis of ASD. Proband diagnosis was confirmed by an expert clinician (T.C.) based on information using the Development and Well-Being Assessment (DAWBA; [23]) and the parent-report Social Communication Questionnaire (SCQ; [24]). Parent-reported family medical histories were examined for significant medical conditions in the proband or extended family members, with no exclusions made on this basis. Infants in the low-risk control group were recruited from a volunteer database. Inclusion criteria included full-term birth, normal birth weight, and lack of any ASD within first-degree family members (as confirmed through parent interview regarding family medical history). All low-risk participants had at least one older sibling. Screening for possible ASD in these older siblings was undertaken using the SCQ, with no child scoring above instrument cut-off for ASD. The data presented in this paper were collected during three consecutive visits, at around 9 months, 15 months, and 2 years of age. All but two low-risk participants and all but two high-risk participants contributed data from at least two visits. General and visit-specific participant characteristics are presented in Table S1.

### Stimuli and Procedure

We created arrays of eight letters, situated on an imaginary circle and on a white background. In each array, all but one stimulus was an “X” letter. The eighth stimulus was either a “+,” a “V,” an “S,” or an “O” (the targets). For each target type, eight different arrays were created, varying in the position of the target, i.e., 32 different stimuli in total. To increase variability, we used letters in an array that were black, blue, red, or green (25% of arrays for each color). Because of time constraints, only 50% of the stimuli were presented at the 2-year-old visit. For each target type (+, V, S, or O), we chose four out of the existing eight stimuli, those where targets were in even area of interest (AOI) positions on the slide (see Figure 1). Infants were seated on mother’s lap, at approximately 60 cm from a Tobii T120 screen. A five-point calibration routine was run. The experiment was started only after at least four points were marked as being properly calibrated for each eye. The infant’s behavior was monitored by a video camera placed above the Tobii monitor. Stimuli were presented with TobiiStudio software. Each of the stimuli was presented once, in a random order, for 1.5 s. Before each stimulus, the child’s attention was directed to the center of the screen using a short audio-video animation. Only trials in which the center of the screen was fixated within the first 100 ms of stimulus onset were used for subsequent analysis.

### Measures of Autism Symptoms

The AOSI [10] is a validated clinical measure of infant risk markers, focusing on precursors of impairments present in the ASD phenotype, including response to name, eye contact, social reciprocity, and imitation. Infant behavior is elicited, while on the parent’s lap, within a structured interaction with an assessor including a series of presses designed to elicit a range of social behaviors. This scale was used to measure autism symptoms at 9 and 15 months, and we report the total score [10]. The ADOS-2 [11] is a semi-structured play-based assessment, used to assess autism-related social and communication behavioral characteristics (all children were administered module 1 of the ADOS-2). This scale was used during the 2-year-old visit, and we report the overall total score, which combines the social affect scale and the repetitive and restrictive behaviors scale.

## Consortia

The members of The BASIS Team are Simon Baron-Cohen, Patrick Bolton, Celeste Cheung, Kim Davies, Michelle Liew, Janice Fernandes, Issy Gammer, Helen Maris, Erica Salomone, Greg Pasco, Andrew Pickles, Helena Ribeiro, and Leslie Tucker. A full list of affiliations for members of The BASIS Team can be found in Table S3.

## Author Contributions

T.G. designed the eye-tracking study. T.G. and The BASIS Team collected the data. T.G. and R.B. analyzed the study and wrote the paper, with contribution from M.H.J. and T.C. M.H.J. and T.C. led The BASIS Team and designed the overall BASIS study, with other members of The BASIS Team.

## Supplemental Information

Document S1. Supplemental Experimental Procedures, Figures S1 and S2, and Tables S1 and S2

Table S3. Member List and Affiliations for The Basis Team

Document S2. Article plus Supplemental Information
