# Supplementary material for: Enhanced Visual Search in Infancy Predicts Emerging Autism Symptoms
Source: Curr Biol. 2015 Jun 29;25(13):1727–30. doi: 10.1016/j.cub.2015.05.011 (PMC4502951; doi:10.1016/j.cub.2015.05.011)
Supplement: Document S1. Supplemental Experimental Procedures, Figures S1 and S2, and Tables S1 and S2 [file mmc1.pdf]

Current Biology
Supplemental Information

Enhanced Visual Search in Infancy Predicts Emerging Autism Symptoms

Teodora Gliga, Rachael Bedford, Tony Charman, Mark H. Johnson, and The BASIS Team

Supplemental Data.

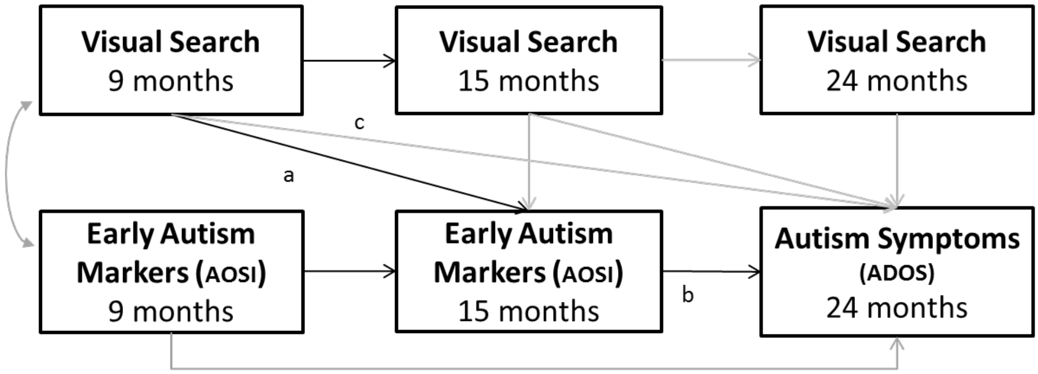

Figure S1. Autoregressive model accounting for early autism symptoms; Related to Figure 2

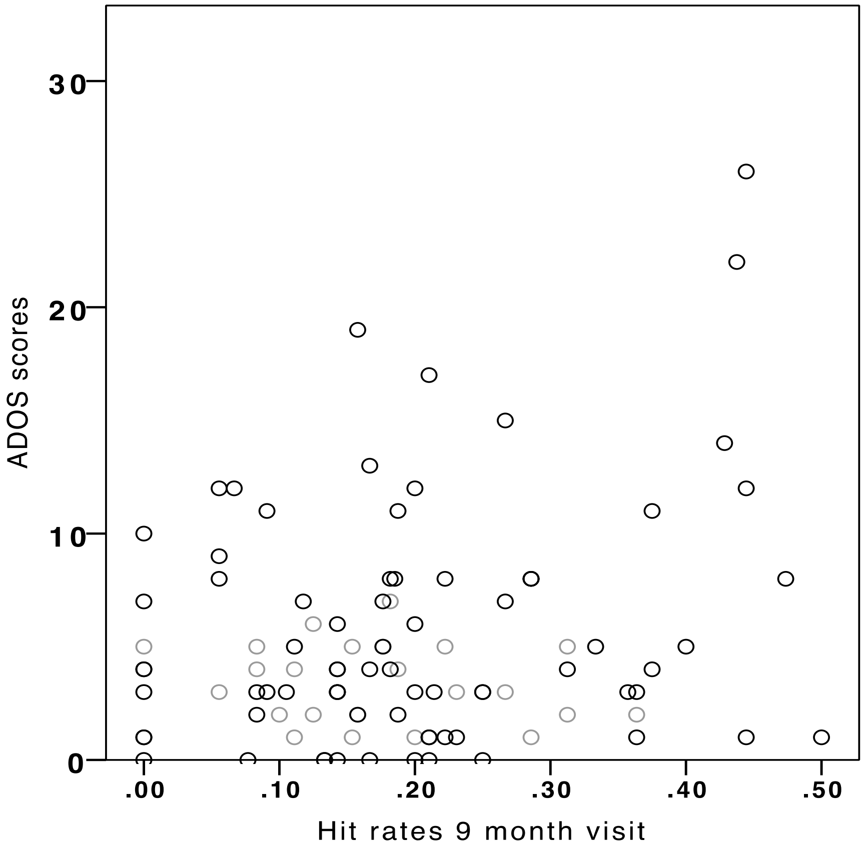

Figure S2. Scatterplot of the relationship between visual search performance at the first visit and autism symptoms at the last visit, for both Low-Risk and High-Risk participants; Related to Figure 2

	Low Risk (n=27)<br>Average (SD; range)	High Risk (n=82)<br>Average (SD; range)
F:M	13:14	37:45
**Age (days)**		
9 months visit	282.7 (25.5; 248-346)	278.6 (25.5; 242-351)
15 months visit	474.0 (27.6; 429-544)	471.8 (30.7; 422-576)
2 years old visit	766.9 (33.4; 734-841)	805.1 (60.8; 734-1052)
**AOSI Score**		
9 months	5.2 (3.1; 1-14)	8.7 (4.7; 0-21)
15 months	4.0 (3.5; 0-15)	6.4 (4.8; 0-22)
**ADOS 2 years** [1]	3.23 (1.7; 1-7)	5.88 (5.5; 0-26)

Table S1. Group characterization. [1]ADOS-2 Overall Total Score (Social Affect + Restricted and Repetitive Behaviour); Related to Experimental Procedure

		Low-Risk for autism	High-Risk for autism
**Visit 1** 9 months	Number valid trials /32	13.50 (3.55)	14.15 (3.98)
	Hit rates	**.17 (.09)**	**.19 (.12)**
	N	24	80
**Visit 2** 15 months	Number valid trials /32	13.2 (4.23)	14.23 (4.74)
	Hit rates	.12 (.11)	**.19 (.11)***
	N	24	71
**Visit 3** 2 years	Number valid trials /16	10.04 (2.9)	10.67 (3.34)
	Hit rates	**.25 (.16)**	**.27 (.17)**
	N	25	69

Table S2. Summary of visual search data. **Bold** font indicates above chance performance, * indicates significant group differences; Related to Results

Supplemental Experimental Procedures
Supplemental Participant Information

Participants took part in a longitudinal study of children at risk for autism. Recruitment, ethical approval and informed consent, as well as background data on participating families, were made available for the current study through BASIS, a UK collaborative network facilitating research with infants at risk for autism. Families enrol when their babies are younger than 5 months of age, and they are invited to attend multiple research visits until their children reach 3 years of age or beyond. At the time of enrolment, none of the infants had been diagnosed with any medical or developmental condition. Twenty-seven Low-Risk participants and 82 High-risk participants took part in this study. The data presented in this paper was collected during 3 consecutive visits, at around 9 months, 15 months and 2 years of age. All but 2 Low-Risk and all but 2 High-Risk participants contributed data from at least two visits. General and visit specific participant characteristics are presented in Table S1.

High-Risk infants had at least one older sibling (hereafter, proband) with a community clinical diagnosis of ASD. 73 probands were male, 9 were female. Proband diagnosis was confirmed by an expert clinician (TC) based on information using the Development and Well Being Assessment (DAWBA; [S1]) and the parent-report Social Communication Questionnaire (SCQ; [S2]). The DAWBA is a parent-completed Web-based assessment that asks parents to rate symptoms of autism, relevant to making *Diagnostic and Statistical Manual of Mental Disorders* (4th ed., text rev.; *DSM–IV–TR*; [S3]) and ICD-10 [S4] diagnosis of autism spectrum disorders. Descriptive information about the child is also included. The expert reviewed the forms using both the scores and the narrative text to assign a diagnosis. The SCQ is a widely used 40-item questionnaire that asks about current and past autism symptoms. Most probands met criteria for ASD on both the DAWBA and SCQ (n = 59). While a small number scored below threshold on the SCQ (n = 7), no exclusions

were made, due to meeting threshold on the DAWBA and expert opinion. For 16 probands, data were only available on one measure (3 DAWBA only, 13 SCQ only), and for all probands at least one measure was available (in addition to parent-confirmed community clinical diagnosis). Parent- reported family medical histories were examined for significant medical conditions in the proband or extended family members, with no exclusions made on this basis. Infants in the Low-risk control group were recruited from a volunteer database. Inclusion criteria included full-term birth, normal birth weight, and lack of any ASD within first-degree family members (as confirmed through parent interview regarding family medical history). All Low-risk participants had at least one older sibling. Screening for possible ASD in these older siblings was undertaken using the SCQ, with no child scoring above instrument cut-off for ASD (1 score missing)

Supplemental methods

Autoregressive models test how the variance-covariance matrix changes over time and are thus ideal for addressing developmental hypotheses. Including autoregressions between the visual search measures at visits 1-3 assumes that performance on visual search at visit 3 can be influenced by performance at visit 1 *only* via visit 2 visual search. Similarly, for the model where there are autoregressions among the symptoms of autism (Figure 2) only indirect effects from 9-month AOSI to 2-year ADOS are specified. Model fit was assessed using the χ^2 test of model fit and the comparative fit index (CFI). The χ^2 test of model fit is an absolute fit statistic, which represents the difference between the unrestricted covariance matrix (the observed data) and the restricted covariance matrix (the model). If this test is *not significant* then we have no evidence to reject the null hypothesis, that there is no difference between the unrestricted and restricted models. In other words the larger the p value, the closer the fit between the data and the model. We have also reported the CFI, which has values ranging from 0 – 1 and for a good model fit values should ideally be above 0.9 [S5].

The analysis was undertaken using Mplus software [S6] and reported results are based on STDYX standardization. For indirect and total effects computation, only p values are reported.

Supplemental Analysis

Autoregressive model accounting for early autism symptoms. To test whether visual search at 9 months continued to predict ADOS score after accounting for earlier autism markers, we ran a autoregressive model with regressions, rather than correlations, between AOSI and ADOS (model fit: $\chi^2(4) = 6.87$, p = 0.14, CFI = 0.95). The relationship between 9-month visual search and 15-month AOSI remained significant (ß = 0.182, S.E. = 0.09, *p* = 0.046). While the *total effect* (see Figure S1; a*b+c) of 9-month visual search on ADOS was significant (p= 0.027), the direct relationship with later ADOS (pathway 'c') (i.e., accounting for 9 and 15 month AOSI) became non-significant (ß = 0.13, S.E. = 0.09, *p* = 0.13). The indirect pathway from 9-month visual search to ADOS via 14-month AOSI was marginally significant (p=0.066), suggesting that the prediction to later ADOS scores is a result, in part, of the relationship to early autism symptoms.

Limitations and future directions.

We demonstrate a relationship between a particular version of a visual search task, one in which letter targets are used, and in which detection of the odd one out element is measured as attention capture and not in an explicit search paradigm. Although it is impossible to tell whether infants were or were not searching for the targets, this design is different from most others used with older children and adults with autism, and which had demonstrated superior ability. Given that continuity in performance in our attention capture task was moderated, and performance was not related to ASD symptoms at 2 years of age, it is possible that the abilities we measured here and in previous studies with older children,

are not reflecting the same underlying mechanisms. In follow-up studies with these infants, we are using classical visual search tasks, and we will be able to investigate the longitudinal relationship between tasks.

Superior performance in ASD was also mainly recorded in conditions in which the search was rendered difficult as for example in conjunction searches, or when target and distracter differ minimally along a single dimension (e.g. [S7], [S8]). We have also used contrasts of varying difficulty (i.e. easy O targets and difficult V targets). The effects we report may well be due to superior performance in the more difficult contrasts (e.g. V/X). Unfortunately, we could not collect enough trials in all conditions to test this hypothesis, which will have to be answered by future studies.

Having used a particular type of stimulus also restricts the generalizability of our results to those features that discriminated between letter targets and foils, e.g. line orientation and curvature or the presence of line crossing. Letters are often used in visual search studies (e.g. [S9]) and children with ASD were shown to perform better at detecting letter shaped targets [S10]. However that may reflect selective superior abilities in processing letter type stimuli, or hyperlexia, which is more common in the ASD population. However, since there is only limited continuity in visual search abilities from 9 to 24 months, we believe it unlikely that visual search is a predictor of later better ability to match or read letters. Nonetheless, it is important for this relationship to be tested and also for our findings to be replicated with other stimulus sets.

Supplemental References

S1. Goodman, R., Ford, T., Richards, H., Gatward, R., & Meltzer, H. (2000). The Development and Well-Being Assessment: description and initial validation of an integrated assessment of child and adolescent psychopathology. *Journal of child psychology and psychiatry*, *41*(05), 645-655.

S2. Rutter, M., Bailey, A., & Lord, C. (2003). *The social communication questionnaire: Manual*. Western Psychological Services.

S3. American Psychiatric Association. (2000). *Diagnostic and statistical manual of mental disorders, text revision (DSM-IV-TR)*. American Psychiatric Association.

S4. World Health Organization. (1993). The ICD-10 Classification of Mental and Behavioural Disorders-Diagnostic Criteria for Research (Geneva, World Health Organization). *J Gambl Stud*.

S5. Bentler, P. M. (1992). On the fit of models to covariances and methodology to the Bulletin. Psychological Bulletin, 112, 400-404.

S6. Muthén, L. K., and Muthén, B. O. (2011). Mplus User's Guide. Sixth Edition. Los Angeles, CA: Muthén & Muthén.

S7. O’riordan, M. A. (2004). Superior visual search in adults with autism. Autism, 8(3), 229-248.

S8. Collignon, O., Charbonneau, G., Peters, F., Nassim, M., Lassonde, M., Lepore, F., Mottron, L. and Bertone, A. (2013). Reduced multisensory facilitation in persons with autism. Cortex, 49(6), 1704-1710.

S9. Treisman, A. M., and Gelade, G. (1980). A feature-integration theory of attention. Cognitive Psychology, 12(1), 97-136.

S10. Jarrold, C., Gilchrist, I. D., and Bender, A. (2005). Embedded figures detection in autism and typical development: Preliminary evidence of a double dissociation in relationships with visual search. Developmental Science, 8(4), 344-351.
