# Supplementary material for: Enhanced Visual Search in Infancy Predicts Emerging Autism Symptoms
Source: Curr Biol. 2015 Jun 29;25(13):1727–30. doi: 10.1016/j.cub.2015.05.011 (PMC4502951; doi:10.1016/j.cub.2015.05.011)
Supplement: Document S2. Article plus Supplemental Information [file mmc3.pdf]

Current Biology

Report

Enhanced Visual Search in Infancy Predicts Emerging Autism Symptoms

Highlights

- We measured visual search abilities in infants at familial risk for autism
- Enhanced visual search at 9 months predicted a higher level of autism symptoms at 2 years
- Atypical perception is intrinsically linked to the emerging autism phenotype

Authors

Teodora Gliga, Rachael Bedford, Tony Charman, Mark H. Johnson, The BASIS Team

Correspondence

t.gliga@bbk.ac.uk

In Brief

It remained unknown whether superior perception, a common feature of the autism phenotype, contributes to the emergence of core social interaction and communication symptoms. Gliga et al. show that superior performance in a visual search task in 9-month-old infants predicts a higher level of autism symptoms at 15 months and 2 years.

Gliga et al., 2015, Current Biology *25*, 1727–1730
June 29, 2015 ©2015 The Authors
http://dx.doi.org/10.1016/j.cub.2015.05.011

Current Biology
Report

Enhanced Visual Search in Infancy Predicts Emerging Autism Symptoms

Teodora Gliga,[1,*] Rachael Bedford,[2] Tony Charman,[3] Mark H. Johnson,[1] and The BASIS Team
[1]Centre for Brain and Cognitive Development, Birkbeck College, University of London, Malet Street, London WC1E 7HX, UK
[2]Department of Biostatistics, Institute of Psychiatry, Psychology and Neuroscience, King's College London, London SE5 8AF, UK
[3]Department of Psychology, Institute of Psychiatry, Psychology and Neuroscience, King's College London, London SE5 8AF, UK
*Correspondence: t.gliga@bbk.ac.uk
http://dx.doi.org/10.1016/j.cub.2015.05.011
This is an open access article under the CC BY license (http://creativecommons.org/licenses/by/4.0/).

SUMMARY

In addition to core symptoms, i.e., social interaction and communication difficulties and restricted and repetitive behaviors, autism is also characterized by aspects of superior perception [1]. One well-replicated finding is that of superior performance in visual search tasks, in which participants have to indicate the presence of an odd-one-out element among a number of foils [2–5]. Whether these aspects of superior perception contribute to the emergence of core autism symptoms remains debated [4, 6]. Perceptual and social interaction atypicalities could reflect co-expressed but biologically independent pathologies, as suggested by a "fractionable" phenotype model of autism [7]. A developmental test of this hypothesis is now made possible by longitudinal cohorts of infants at high risk, such as of younger siblings of children with autism spectrum disorder (ASD). Around 20% of younger siblings are diagnosed with autism themselves [8], and up to another 30% manifest elevated levels of autism symptoms [9]. We used eye tracking to measure spontaneous orienting to letter targets (O, S, V, and +) presented among distractors (the letter X; Figure 1). At 9 and 15 months, emerging autism symptoms were assessed using the Autism Observation Scale for Infants (AOSI; [10]), and at 2 years of age, they were assessed using the Autism Diagnostic Observation Schedule (ADOS; [11]). Enhanced visual search performance at 9 months predicted a higher level of autism symptoms at 15 months and at 2 years. Infant perceptual atypicalities are thus intrinsically linked to the emerging autism phenotype.

RESULTS AND DISCUSSION

Eighty-two high-risk infants (37 girls) and 27 low-risk controls (13 girls) took part in this study (Table S1). We analyzed the proportion of trials in which infants made a first look toward one of the targets, after fixating at the center of the screen. Infants with at least four valid trials were included in the analysis. Above-chance performance was measured in the group as a whole, at all ages (9 months: $t(103) = 5.62$, $p < 0.001$; 15 months: $t(95) = 4.31$, $p < 0.001$; 2 years: $t(94) = 7.9$, $p < 0.001$; Table S2). Performance was not related to either the age or IQ of the participant (all $p > 0.09$) during any of the visits.

A shift from categorical to continuous characterization of psychopathology is encouraged by clinical and genetics research [12, 13]. Thus, to take into account longitudinal relationships between visual search performance and emerging autism symptoms (Figure 1), we entered search performance at 9 months, 15 months, and 2 years in an autoregressive model (Figure 2; model fit: $\chi^2(4) = 6.87$, $p = 0.14$, comparative fit index [CFI] = 0.95) with continuous measures of symptom severity at 9 and 15 months (Autism Observation Scale for Infants [AOSI] score) and 2 years of age (Autism Diagnostic Observation Schedule [ADOS] score). Nine-month visual search significantly predicted the 15-month AOSI ($\beta = 0.22$, SE = 0.10, $p = 0.03$) and the 2-year ADOS ($\beta = 0.24$, SE = 0.10, $p = 0.02$; see also Figure S2) score, with increased visual search accuracy predicting higher symptom severity. Findings were very similar when only the high-risk group was included in the analysis (model fit: $\chi^2(4) = 10.001$, $p = 0.04$, CFI = 0.88). The relationship with ADOS remained substantively similar: 9-month visual search was significantly related to both 15-month AOSI ($\beta = 0.223$, SE = 0.11, $p = 0.049$) and 2-year ADOS ($\beta = 0.27$, SE = 0.11, $p = 0.02$) scores. To test whether visual search at 9 months continued to predict ADOS score after accounting for earlier autism markers, we ran an autoregressive model with regressions, rather than correlations, between AOSI and ADOS (model fit: $\chi^2(4) = 6.87$, $p = 0.14$, CFI = 0.95; Figure S1). The relationship between 9-month visual search and 15-month AOSI remained significant ($\beta = 0.182$, SE = 0.09, $p = 0.046$), but the direct relationship with later ADOS (i.e., accounting for 9- and 15-month AOSI) became nonsignificant ($\beta = 0.13$, SE = 0.09, $p = 0.13$), suggesting a developmental pathway in which infant visual search contributes to autism symptoms at 15 months of age and that in turn contributes to autism severity at 2 years of age. The lack of a concurrent relationship between visual search and symptom severity at 9 months ($\beta = 0.08$, SE = 0.10, $p = 0.44$) is suggestive of a causal pathway from early perception to later emerging autism symptoms. Moreover, although 9- and 15-month visual search performances are correlated ($\beta = 0.24$, SE = 0.10, $p = 0.02$), performance at later time points, i.e., at 15 months and 2 years of age, does not relate to symptomatology. This differential relationship points to particular periods in early postnatal development within which atypical perception, in addition to other risk factors, may set development on a pathway to pathology and

CellPress

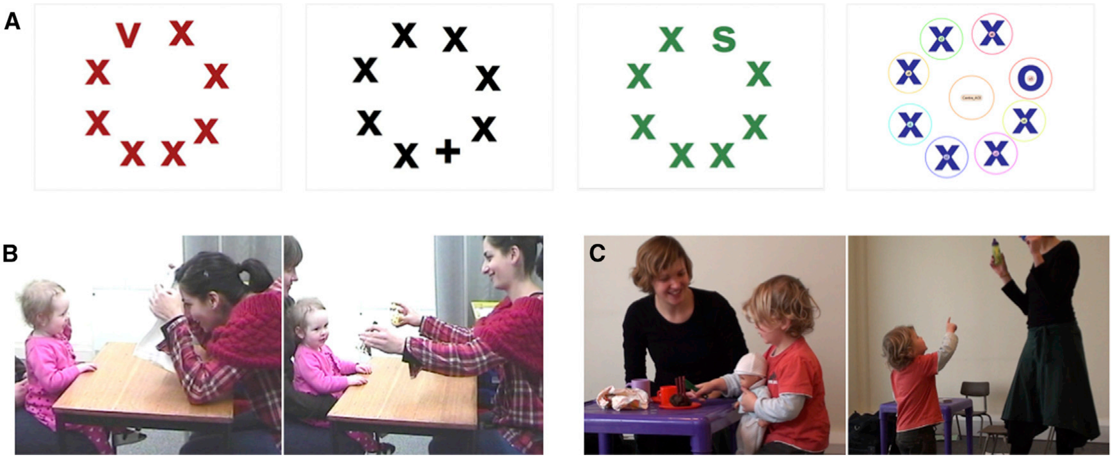

Figure 1. Study Design, Stimuli, and Behavioral Assessments
(A) Example stimuli and the areas of interest (AOIs) used in analysis.
(B) Example of behaviors assessed with the AOSI (e.g., anticipation of social contact, attention shifting).
(C) Example of behaviors assessed with the ADOS (e.g., pretend play, pointing).

further highlights the importance of prospective studies of early development.

We demonstrate, for the first time, a relationship between superior visual search abilities during infancy and the severity of later autism symptoms. Because we analyzed the first saccade made in each trial, and not whether infants ever visited the target during the trial (as in some previous visual search studies [5]), we are confident our findings are not confounded by differences in oculomotor behavior described in this population [14]. Also, given a higher incidence of hyperlexia in autism [15], future studies should address the question of whether the demonstrated superior visual search also predicts better recognition of letters later in childhood.

Views on atypical perception have alternated between assigning it a core, causal role in autism [16] and portraying it as "one aspect of cognition in autism spectrum disorder (ASD) alongside, rather than causing/explaining, deficits in social cognition" [6]. Importantly, our findings corroborate evidence for atypical oculomotor behavior [14] and increased frontal-occipital functional connectivity [17] during the first year of life of those infants that later develop autism symptoms, by suggesting that perturbations in general processes, such as perception or attention, are more important than previously believed in the developmental pathway to this disorder [18]. With this shift away from "social brain" theories of autism (e.g., [19]) comes also the challenge of explaining the mechanisms through which domain-general atypicalities could contribute to the emergence of specific autism symptoms. Moreover, the striking predictive association between superior visual search and autism may also prove useful as one additional component of early autism identification, given a context in which most current infant markers are based on impairments common to multiple neurodevelopmental outcomes (e.g., [20–22]).

EXPERIMENTAL PROCEDURES

Participants

Participants took part in a longitudinal study of children at risk for autism. At the time of enrollment, none of the infants had been diagnosed with any medical or developmental condition. Twenty-seven low-risk participants and 82 high-risk participants took part in this study. High-risk infants had at least one older sibling (hereafter, proband) with a community clinical diagnosis of ASD. Proband diagnosis was confirmed by an expert clinician (T.C.) based on information using the Development and Well-Being Assessment (DAWBA; [23]) and the parent-report Social Communication Questionnaire (SCQ; [24]). Parent-reported family medical histories were examined for significant medical conditions in the proband or extended family members, with no exclusions made on this basis. Infants in the low-risk control group were recruited from a volunteer database. Inclusion criteria included full-term birth, normal birth weight, and lack of any ASD within first-degree family members (as confirmed through parent interview regarding family medical history). All low-risk participants had at least one older sibling. Screening for possible ASD in these older siblings was undertaken using the SCQ, with no child scoring above instrument cut-off for ASD. The data presented in this paper were collected during three consecutive visits, at around 9 months, 15 months, and 2 years of age. All but two low-risk participants and all but two high-risk participants contributed data from at least two visits. General and visit-specific participant characteristics are presented in Table S1.

Stimuli and Procedure

We created arrays of eight letters, situated on an imaginary circle and on a white background. In each array, all but one stimulus was an "X" letter. The eighth stimulus was either a "+," a "V," an "S," or an "O" (the targets). For each target type, eight different arrays were created, varying in the position of the target, i.e., 32 different stimuli in total. To increase variability, we used letters in an array that were black, blue, red, or green (25% of arrays for each color). Because of time constraints, only 50% of the stimuli were presented at the 2-year-old visit. For each target type (+, V, S, or O), we chose four out of the existing eight stimuli, those where targets were in even area of interest (AOI) positions on the slide (see Figure 1). Infants were seated on mother's lap, at approximately 60 cm from a Tobii T120 screen. A five-point calibration routine was run. The experiment was started only after at least four points were marked as being properly calibrated for each eye. The infant's behavior was monitored by a video camera placed above the Tobii monitor. Stimuli were presented with TobiiStudio software. Each of the stimuli was presented once, in a random order, for 1.5 s. Before each stimulus, the child's attention was directed to the center of the screen using a short audio-video animation. Only trials in which the center of the screen was fixated within the first 100 ms of stimulus onset were used for subsequent analysis.

Measures of Autism Symptoms

The AOSI [10] is a validated clinical measure of infant risk markers, focusing on precursors of impairments present in the ASD phenotype, including response

CellPress

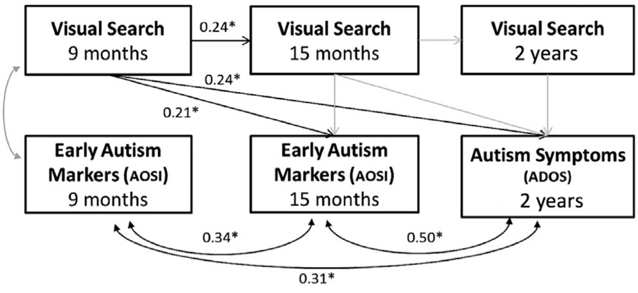

Figure 2. Relationship between Visual Search and Emerging Autism Symptoms

Visual search performance at 9 months predicts later autism symptom severity in an autoregressive model. Standardized coefficient values are presented for significant results (represented as black arrows).

to name, eye contact, social reciprocity, and imitation. Infant behavior is elicited, while on the parent's lap, within a structured interaction with an assessor including a series of presses designed to elicit a range of social behaviors. This scale was used to measure autism symptoms at 9 and 15 months, and we report the total score [10]. The ADOS-2 [11] is a semi-structured play-based assessment, used to assess autism-related social and communication behavioral characteristics (all children were administered module 1 of the ADOS-2). This scale was used during the 2-year-old visit, and we report the overall total score, which combines the social affect scale and the repetitive and restrictive behaviors scale.

SUPPLEMENTAL INFORMATION

Supplemental Information includes Supplemental Experimental Procedures, two figures, and three tables and can be found with this article online at http://dx.doi.org/10.1016/j.cub.2015.05.011.

CONSORTIA

The members of The BASIS Team are Simon Baron-Cohen, Patrick Bolton, Celeste Cheung, Kim Davies, Michelle Liew, Janice Fernandes, Issy Gammer, Helen Maris, Erica Salomone, Greg Pasco, Andrew Pickles, Helena Ribeiro, and Leslie Tucker. A full list of affiliations for members of The BASIS Team can be found in Table S3.

AUTHOR CONTRIBUTIONS

T.G. designed the eye-tracking study. T.G. and The BASIS Team collected the data. T.G. and R.B. analyzed the study and wrote the paper, with contribution from M.H.J. and T.C. M.H.J. and T.C. led The BASIS Team and designed the overall BASIS study, with other members of The BASIS Team.

ACKNOWLEDGMENTS

Thanks to all the families who gave up their time to participate in the studies. This work was supported by MRC Programme Grant no. G0701484, the BASIS funding consortium led by Autistica, a Sir Henry Wellcome Postdoctoral Fellowship to R.B., ESF COST Action BM1004, and EU-AIMS (the Innovative Medicines Initiative joint undertaking grant agreement no. 115300, resources of which are composed of financial contributions from the European Union's Seventh Framework Programme [FP7/2007-2013] and EFPIA companies' in-kind contribution).

Received: March 16, 2015
Revised: April 20, 2015
Accepted: May 6, 2015
Published: June 11, 2015

REFERENCES

1. Mottron, L., Dawson, M., Soulières, I., Hubert, B., and Burack, J. (2006). Enhanced perceptual functioning in autism: an update, and eight principles of autistic perception. J. Autism Dev. Disord. *36*, 27–43.
2. Plaisted, K., O'Riordan, M., and Baron-Cohen, S. (1998). Enhanced visual search for a conjunctive target in autism: a research note. J. Child Psychol. Psychiatry *39*, 777–783.
3. Kemner, C., van Ewijk, L., van Engeland, H., and Hooge, I. (2008). Brief report: eye movements during visual search tasks indicate enhanced stimulus discriminability in subjects with PDD. J. Autism Dev. Disord. *38*, 553–557.
4. Jarrold, C., Gilchrist, I.D., and Bender, A. (2005). Embedded figures detection in autism and typical development: preliminary evidence of a double dissociation in relationships with visual search. Dev. Sci. *8*, 344–351.
5. Kaldy, Z., Kraper, C., Carter, A.S., and Blaser, E. (2011). Toddlers with Autism Spectrum Disorder are more successful at visual search than typically developing toddlers. Dev. Sci. *14*, 980–988.
6. Happé, F., and Frith, U. (2006). The weak coherence account: detail-focused cognitive style in autism spectrum disorders. J. Autism Dev. Disord. *36*, 5–25.
7. Happé, F., and Ronald, A. (2008). The 'fractionable autism triad': a review of evidence from behavioural, genetic, cognitive and neural research. Neuropsychol. Rev. *18*, 287–304.
8. Ozonoff, S., Young, G.S., Carter, A., Messinger, D., Yirmiya, N., Zwaigenbaum, L., Bryson, S., Carver, L.J., Constantino, J.N., Dobkins, K., et al. (2011). Recurrence risk for autism spectrum disorders: a Baby Siblings Research Consortium study. Pediatrics *128*, e488–e495.
9. Messinger, D., Young, G.S., Ozonoff, S., Dobkins, K., Carter, A., Zwaigenbaum, L., Landa, R.J., Charman, T., Stone, W.L., Constantino, J.N., et al. (2013). Beyond autism: a baby siblings research consortium study of high-risk children at three years of age. J. Am. Acad. Child Adolesc. Psychiatry *52*, 300–308, e1.
10. Bryson, S.E., Zwaigenbaum, L., McDermott, C., Rombough, V., and Brian, J. (2008). The Autism Observation Scale for Infants: scale development and reliability data. J. Autism Dev. Disord. *38*, 731–738.
11. Lord, C., Rutter, M., DiLavore, P., Risi, S., Gotham, K., and Bishop, S. (2012). Autism Diagnostic Observation Schedule, Second Edition (ADOS-2). (Los Angeles: Western Psychological Services).
12. American Psychiatric Association (2013). Diagnostic and Statistical Manual of Mental Disorders, Fifth Edition. (American Psychiatric Publishing).
13. Plomin, R., Haworth, C.M., and Davis, O.S. (2009). Common disorders are quantitative traits. Nat. Rev. Genet. *10*, 872–878.
14. Wass, S.V., Jones, E.J., Gliga, T., Smith, T.J., Charman, T., and Johnson, M.H.; BASIS team (2015). Shorter spontaneous fixation durations in infants with later emerging autism. Sci Rep *5*, 8284.
15. Grigorenko, E.L., Klin, A., and Volkmar, F. (2003). Annotation: Hyperlexia: disability or superability? J. Child Psychol. Psychiatry *44*, 1079–1091.
16. Frith, U. (1989). Autism: Explaining the Enigma, Second Edition. (Wiley-Blackwell).
17. Orekhova, E.V., Elsabbagh, M., Jones, E.J., Dawson, G., Charman, T., and Johnson, M.H.; BASIS Team (2014). EEG hyper-connectivity in high-risk infants is associated with later autism. J Neurodev Disord *6*, 40.
18. Gliga, T., Jones, E.J., Bedford, R., Charman, T., and Johnson, M.H. (2014). From early markers to neuro-developmental mechanisms of autism. Dev. Rev. *34*, 189–207.
19. Johnson, M.H. (2014). Autism: demise of the innate social orienting hypothesis. Curr. Biol. *24*, R30–R31.

CellPress

20. Jones, W., and Klin, A. (2013). Attention to eyes is present but in decline in 2-6-month-old infants later diagnosed with autism. Nature *504*, 427–431.
21. Sacrey, L.A.R., Bryson, S.E., and Zwaigenbaum, L. (2013). Prospective examination of visual attention during play in infants at high-risk for autism spectrum disorder: a longitudinal study from 6 to 36 months of age. Behav. Brain Res. *256*, 441–450.
22. Elsabbagh, M., Mercure, E., Hudry, K., Chandler, S., Pasco, G., Charman, T., Pickles, A., Baron-Cohen, S., Bolton, P., and Johnson, M.H.; BASIS Team (2012). Infant neural sensitivity to dynamic eye gaze is associated with later emerging autism. Curr. Biol. *22*, 338–342.
23. Goodman, R., Ford, T., Richards, H., Gatward, R., and Meltzer, H. (2000). The Development and Well-Being Assessment: description and initial validation of an integrated assessment of child and adolescent psychopathology. J. Child Psychol. Psychiatry *41*, 645–655.
24. Rutter, M., Bailey, A., and Lord, C. (2003). The Social Communication Questionnaire: Manual. (Western Psychological Services).

Current Biology

Supplemental Information

Enhanced Visual Search in Infancy Predicts Emerging Autism Symptoms

Teodora Gliga, Rachael Bedford, Tony Charman, Mark H. Johnson, and The BASIS Team

Supplemental Data.

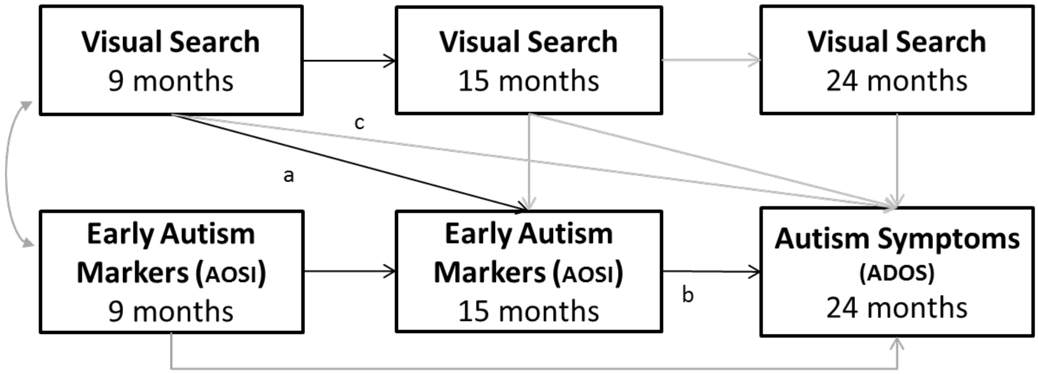

Figure S1. Autoregressive model accounting for early autism symptoms; Related to Figure 2

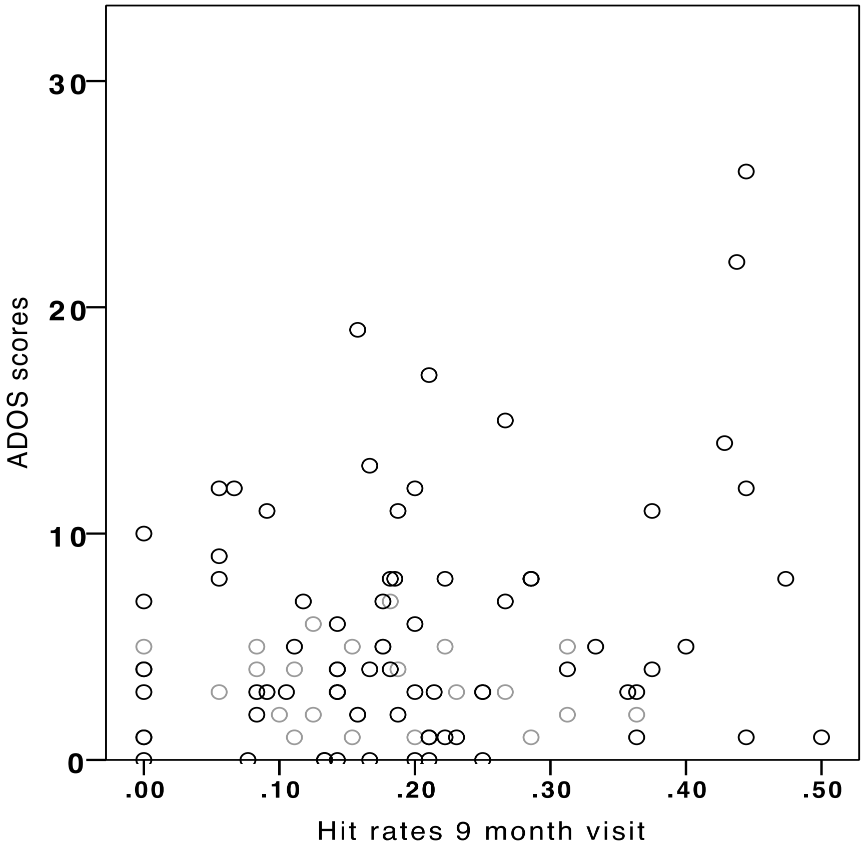

Figure S2. Scatterplot of the relationship between visual search performance at the first visit and autism symptoms at the last visit, for both Low-Risk and High-Risk participants; Related to Figure 2

	Low Risk (n=27)<br>Average (SD; range)	High Risk (n=82)<br>Average (SD; range)
F:M	13:14	37:45
**Age (days)**		
9 months visit	282.7 (25.5; 248-346)	278.6 (25.5; 242-351)
15 months visit	474.0 (27.6; 429-544)	471.8 (30.7; 422-576)
2 years old visit	766.9 (33.4; 734-841)	805.1 (60.8; 734-1052)
**AOSI Score**		
9 months	5.2 (3.1; 1-14)	8.7 (4.7; 0-21)
15 months	4.0 (3.5; 0-15)	6.4 (4.8; 0-22)
**ADOS 2 years** [1]	3.23 (1.7; 1-7)	5.88 (5.5; 0-26)

Table S1. Group characterization. [1]ADOS-2 Overall Total Score (Social Affect + Restricted and Repetitive Behaviour); Related to Experimental Procedure

		Low-Risk for autism	High-Risk for autism
**Visit 1** 9 months	Number valid trials /32	13.50 (3.55)	14.15 (3.98)
	Hit rates	**.17 (.09)**	**.19 (.12)**
	N	24	80
**Visit 2** 15 months	Number valid trials /32	13.2 (4.23)	14.23 (4.74)
	Hit rates	.12 (.11)	**.19 (.11)***
	N	24	71
**Visit 3** 2 years	Number valid trials /16	10.04 (2.9)	10.67 (3.34)
	Hit rates	**.25 (.16)**	**.27 (.17)**
	N	25	69

Table S2. Summary of visual search data. **Bold** font indicates above chance performance, * indicates significant group differences; Related to Results

Supplemental Experimental Procedures
Supplemental Participant Information

Participants took part in a longitudinal study of children at risk for autism. Recruitment, ethical approval and informed consent, as well as background data on participating families, were made available for the current study through BASIS, a UK collaborative network facilitating research with infants at risk for autism. Families enrol when their babies are younger than 5 months of age, and they are invited to attend multiple research visits until their children reach 3 years of age or beyond. At the time of enrolment, none of the infants had been diagnosed with any medical or developmental condition. Twenty-seven Low-Risk participants and 82 High-risk participants took part in this study. The data presented in this paper was collected during 3 consecutive visits, at around 9 months, 15 months and 2 years of age. All but 2 Low-Risk and all but 2 High-Risk participants contributed data from at least two visits. General and visit specific participant characteristics are presented in Table S1.

High-Risk infants had at least one older sibling (hereafter, proband) with a community clinical diagnosis of ASD. 73 probands were male, 9 were female. Proband diagnosis was confirmed by an expert clinician (TC) based on information using the Development and Well Being Assessment (DAWBA; [S1]) and the parent-report Social Communication Questionnaire (SCQ; [S2]). The DAWBA is a parent-completed Web-based assessment that asks parents to rate symptoms of autism, relevant to making *Diagnostic and Statistical Manual of Mental Disorders* (4th ed., text rev.; *DSM–IV–TR*; [S3]) and ICD-10 [S4] diagnosis of autism spectrum disorders. Descriptive information about the child is also included. The expert reviewed the forms using both the scores and the narrative text to assign a diagnosis. The SCQ is a widely used 40-item questionnaire that asks about current and past autism symptoms. Most probands met criteria for ASD on both the DAWBA and SCQ (n = 59). While a small number scored below threshold on the SCQ (n = 7), no exclusions

were made, due to meeting threshold on the DAWBA and expert opinion. For 16 probands, data were only available on one measure (3 DAWBA only, 13 SCQ only), and for all probands at least one measure was available (in addition to parent-confirmed community clinical diagnosis). Parent- reported family medical histories were examined for significant medical conditions in the proband or extended family members, with no exclusions made on this basis. Infants in the Low-risk control group were recruited from a volunteer database. Inclusion criteria included full-term birth, normal birth weight, and lack of any ASD within first-degree family members (as confirmed through parent interview regarding family medical history). All Low-risk participants had at least one older sibling. Screening for possible ASD in these older siblings was undertaken using the SCQ, with no child scoring above instrument cut-off for ASD (1 score missing)

Supplemental methods

Autoregressive models test how the variance-covariance matrix changes over time and are thus ideal for addressing developmental hypotheses. Including autoregressions between the visual search measures at visits 1-3 assumes that performance on visual search at visit 3 can be influenced by performance at visit 1 *only* via visit 2 visual search. Similarly, for the model where there are autoregressions among the symptoms of autism (Figure 2) only indirect effects from 9-month AOSI to 2-year ADOS are specified. Model fit was assessed using the χ^2 test of model fit and the comparative fit index (CFI). The χ^2 test of model fit is an absolute fit statistic, which represents the difference between the unrestricted covariance matrix (the observed data) and the restricted covariance matrix (the model). If this test is *not significant* then we have no evidence to reject the null hypothesis, that there is no difference between the unrestricted and restricted models. In other words the larger the p value, the closer the fit between the data and the model. We have also reported the CFI, which has values ranging from 0 – 1 and for a good model fit values should ideally be above 0.9 [S5].

The analysis was undertaken using Mplus software [S6] and reported results are based on STDYX standardization. For indirect and total effects computation, only p values are reported.

Supplemental Analysis

Autoregressive model accounting for early autism symptoms. To test whether visual search at 9 months continued to predict ADOS score after accounting for earlier autism markers, we ran a autoregressive model with regressions, rather than correlations, between AOSI and ADOS (model fit: $\chi^2(4) = 6.87$, p = 0.14, CFI = 0.95). The relationship between 9-month visual search and 15-month AOSI remained significant (ß = 0.182, S.E. = 0.09, *p* = 0.046). While the *total effect* (see Figure S1; a*b+c) of 9-month visual search on ADOS was significant (p= 0.027), the direct relationship with later ADOS (pathway 'c') (i.e., accounting for 9 and 15 month AOSI) became non-significant (ß = 0.13, S.E. = 0.09, *p* = 0.13). The indirect pathway from 9-month visual search to ADOS via 14-month AOSI was marginally significant (p=0.066), suggesting that the prediction to later ADOS scores is a result, in part, of the relationship to early autism symptoms.

Limitations and future directions.

We demonstrate a relationship between a particular version of a visual search task, one in which letter targets are used, and in which detection of the odd one out element is measured as attention capture and not in an explicit search paradigm. Although it is impossible to tell whether infants were or were not searching for the targets, this design is different from most others used with older children and adults with autism, and which had demonstrated superior ability. Given that continuity in performance in our attention capture task was moderated, and performance was not related to ASD symptoms at 2 years of age, it is possible that the abilities we measured here and in previous studies with older children,

are not reflecting the same underlying mechanisms. In follow-up studies with these infants, we are using classical visual search tasks, and we will be able to investigate the longitudinal relationship between tasks.

Superior performance in ASD was also mainly recorded in conditions in which the search was rendered difficult as for example in conjunction searches, or when target and distracter differ minimally along a single dimension (e.g. [S7], [S8]). We have also used contrasts of varying difficulty (i.e. easy O targets and difficult V targets). The effects we report may well be due to superior performance in the more difficult contrasts (e.g. V/X). Unfortunately, we could not collect enough trials in all conditions to test this hypothesis, which will have to be answered by future studies.

Having used a particular type of stimulus also restricts the generalizability of our results to those features that discriminated between letter targets and foils, e.g. line orientation and curvature or the presence of line crossing. Letters are often used in visual search studies (e.g. [S9]) and children with ASD were shown to perform better at detecting letter shaped targets [S10]. However that may reflect selective superior abilities in processing letter type stimuli, or hyperlexia, which is more common in the ASD population. However, since there is only limited continuity in visual search abilities from 9 to 24 months, we believe it unlikely that visual search is a predictor of later better ability to match or read letters. Nonetheless, it is important for this relationship to be tested and also for our findings to be replicated with other stimulus sets.

Supplemental References

S1. Goodman, R., Ford, T., Richards, H., Gatward, R., & Meltzer, H. (2000). The Development and Well-Being Assessment: description and initial validation of an integrated assessment of child and adolescent psychopathology. *Journal of child psychology and psychiatry*, *41*(05), 645-655.

S2. Rutter, M., Bailey, A., & Lord, C. (2003). *The social communication questionnaire: Manual*. Western Psychological Services.

S3. American Psychiatric Association. (2000). *Diagnostic and statistical manual of mental disorders, text revision (DSM-IV-TR)*. American Psychiatric Association.

S4. World Health Organization. (1993). The ICD-10 Classification of Mental and Behavioural Disorders-Diagnostic Criteria for Research (Geneva, World Health Organization). *J Gambl Stud*.

S5. Bentler, P. M. (1992). On the fit of models to covariances and methodology to the Bulletin. Psychological Bulletin, 112, 400-404.

S6. Muthén, L. K., and Muthén, B. O. (2011). Mplus User's Guide. Sixth Edition. Los Angeles, CA: Muthén & Muthén.

S7. O'riordan, M. A. (2004). Superior visual search in adults with autism. Autism, 8(3), 229-248.

S8. Collignon, O., Charbonneau, G., Peters, F., Nassim, M., Lassonde, M., Lepore, F., Mottron, L. and Bertone, A. (2013). Reduced multisensory facilitation in persons with autism. Cortex, 49(6), 1704-1710.

S9. Treisman, A. M., and Gelade, G. (1980). A feature-integration theory of attention. Cognitive Psychology, 12(1), 97-136.

S10. Jarrold, C., Gilchrist, I. D., and Bender, A. (2005). Embedded figures detection in autism and typical development: Preliminary evidence of a double dissociation in relationships with visual search. Developmental Science, 8(4), 344-351.
